# ADAR editing in viruses: an evolutionary force to reckon with

**DOI:** 10.1093/gbe/evab240

**Published:** 2021-10-25

**Authors:** Helen Piontkivska, Benjamin Wales-McGrath, Michael Miyamoto, Marta L Wayne

**Affiliations:** 1 Department of Biological Sciences, 256 Cunningham Hall, Kent State University, Kent, OH, USA 44242; 2 School of Biomedical Sciences, Kent State University, PO Box 5190, Kent, OH, USA 44242; 3 Brain Health Research Institute, Kent State University, Kent, OH, USA 44242; 4 Department of Biology, University of Florida, Gainesville, FL, USA 32611

**Keywords:** RNA editing, adenosine deaminases acting on RNA (ADAR) editing, viral molecular evolution, A-to-I editing, immune response, interferon, RNA viruses, Zika virus, SARS-CoV-2

## Abstract

Adenosine Deaminases that Act on RNA (ADARs) are RNA editing enzymes that play a dynamic and nuanced role in regulating transcriptome and proteome diversity. This editing can be highly selective, affecting a specific site within a transcript, or nonselective, resulting in hyperediting. ADAR editing is important for regulating neural functions and autoimmunity, and has a key role in the innate immune response to viral infections, where editing can have a range of pro- or antiviral effects and can contribute to viral evolution. Here we examine the role of ADAR editing across a broad range of viral groups. We propose that the effect of ADAR editing on viral replication, whether pro- or antiviral, is better viewed as an axis rather than a binary, and that the specific position of a given virus on this axis is highly dependent on virus- and host-specific factors, and can change over the course of infection. However, more research needs to be devoted to understanding these dynamic factors and how they affect virus-ADAR interactions and viral evolution. Another area which warrants significant attention is the effect of virus-ADAR interactions on host-ADAR interactions, particularly in light of the crucial role of ADAR in regulating neural functions. Answering these questions will be essential to developing our understanding of the relationship between ADAR editing and viral infection. In turn, this will further our understanding of the effects of viruses such as SARS-CoV-2, as well as many others, and thereby influence our approach to treating these deadly diseases.

**Significance statement:**

ADAR editing plays an important role in dynamic gene regulation in animals and is a key element of the innate immune response against viral infections. Effects of editing have been documented only in a few groups of viruses, including some examples where editing contributes to viral evolution. Here we summarize available evidence of virus-ADAR interactions across multiple groups of viruses, with primary focus on RNA viruses, including two recent threats, Zika virus and SARS-CoV-2. We call attention to the need for more research on virus-ADAR interactions in light of the role that viral-activated ADARs may play in pathogenesis, including the neurological consequences of viral infections.

*Competing interests*

The authors declare that they have no competing interests.

*Funding*

This work was partially supported by a Brain Health Research Institute Pilot Award and Healthy Communities Research Initiative Launch Pad Award from Kent State University, and the National Institutes of Health (NIA award R21AG064479-01 and RF1AG054654). The funders had no role in the design of the study and collection, analysis, and interpretation of data or in writing the manuscript.

*Authors' contributions*

HP, MM, and MLW conceived the review and wrote the manuscript. BWM contributed to aggregating the data and writing the manuscript. All authors read and approved the final manuscript.

*Acknowledgements*

Not applicable.

## Introduction:

The ongoing pandemic caused by SARS-CoV-2, a novel coronavirus that emerged in late 2019 (Andersen et al. 2020; Zhang and Holmes 2020), has already resulted in 178+ million confirmed cases and 3.8+ million deaths worldwide (WHO Dashboard as of June 22, 2021). However, during the past decade there have been multiple other major public health emergencies due to known or emergent RNA viruses, such as the Ebola (EBOV) and Zika (ZIKV) viruses, bringing to the forefront yet again the necessity to better understand the biology of RNA viruses (Holmes 2009) and the role of inherent properties of their genomes - such as high rates of molecular evolution (Dolan et al. 2018) and resultant genome instabilities (Kockler and Gordenin 2021) - in both the emergence of novel pathogens and the ongoing host-virus interactions resulting in disease and viral spread.

Due to error-prone RNA-dependent RNA polymerase (RdRp), RNA viruses have mutation rates higher than those of other viruses (Duffy et al. 2008; Holmes 2009). Moreover, the substitutions introduced by host editing enzymes (APOBECs and ADARs, Apolipoprotein B mRNA-Editing Catalytic polypeptide-like and Adenosine Deaminases that Act on RNA, respectively) play a non-trivial role in viral evolution, acting as a supplementary source of mutations (Dolan et al. 2018) that may potentially lead to immune escape or treatment resistance (e.g., in measles virus (Patterson et al. 2001)). On the other hand, viral editing by deaminases has been extensively documented as an important component of antiviral innate response in different animal hosts (reviewed in Tomaselli et al. (2015) and Pfaller et al. (2021)). While the details of ADAR and APOBEC editing in viruses and their physiological and evolutionary consequences are still being elucidated, the available evidence points to nuanced interactions between editing and specific viruses. For example, the proviral or antiviral role of editing (Samuel 2011, 2012) may change depending on the stage of the infection (e.g., early versus late in Zika virus infection (Piontkivska et al. 2017; Zhou et al. 2019)). Note that here we interpret “proviral” in the narrow sense of specific nucleotide changes resulting in an increase in viral fitness, rather than a broader interpretation where ADARs’ inhibition of other parts of the immune response facilitates viral replication or decreases interferon (IFN) response (e.g., Lamers et al. 2019; Okonski and Samuel 2013). Further, the consequences of viral editing on the host biology, including the potential for long-term (unintended) changes to host transcriptomes due to dysregulation of editing, remains to be understood (Piontkivska et al. 2019).

ADAR editing is of particular prominence when we consider host-virus interactions because of ADARs’ dual role as major and, in some cases, essential gene expression regulators of nervous system genes (Eisenberg and Levanon 2018). In this review we will focus on the role of ADAR editing across major groups of RNA viruses, highlighting examples where ADAR editing has been documented, and pointing out groups of viruses where editing is likely to occur but has not yet been documented. ADAR editing in DNA viruses has also been reported, e.g., in polyomavirus (MpyV) (George and Samuel 2011) and in herpesviruses, where site-specific editing in EBV (Iizasa et al. 2010; Lei et al. 2013) exemplifies a post-transcriptional modulation of expression and activity of both viral and host miRNAs (Tomaselli et al. 2015). The signature of ADAR hyperediting was also reported in the novel 'near the transcription origin' (NTO) transcript NTO3 in another herpesvirus, varicella zoster virus (VZV), where it was thought to play a role in avoiding degradation, although further studies are needed (Prazsák et al. 2018).

**What Are ADARs?**

Since the discovery of A-to-I (adenosine to inosine) RNA modification in the late 1980s (Bass and Weintraub 1988; Samuel 2019), we have learned about many roles of these "edits", ranging from their key role in innate immune responses (Bass 2002; Samuel 2011) to multiple mechanisms of dynamic post-transcriptional regulation of the transcriptome and proteome diversity, from codon recoding to splicing regulation to miRNA biogenesis (Chen 2013; Nishikura 2016; Eisenberg and Levanon 2018; Michlewski and Caceres 2019). Enzymes from the ADAR (Adenosine Deaminases that Act on RNA) gene family are RNA editing enzymes that deaminate adenosines (A) to produce inosines (I) within double-stranded RNAs (dsRNAs) (Bass 2002), leading to A-to-I changes, referred to as ADAR editing. These A-to-I changes are interpreted as A-to-G substitutions by the cellular machinery, including during translation, thus modulating proteome diversity and expression by potentially introducing nonsynonymous (amino acid changing) substitutions or splice site changes, and serving as a mechanism of dynamic and nuanced post-transcriptional regulation of gene expression (Deffit and Hundley 2016; Walkley et al. 2012). In viral genomes, these changes are likewise converted to A-to-G by viral polymerases during RNA-dependent RNA replication (Pfaller et al. 2011). Moreover, A-to-I edits can change the stability of the dsRNA structures due to the weaker strength of the I-C bond (Bass 2002). For consistency, here we use A-to-G (and complementary U-to-C) nucleotide changes to denote ADAR editing and hyperediting, albeit it should be noted that some original studies refer to these changes as A-to-I instead of A-to-G.

In mammalian genomes there are three ADAR loci, of which two encode the enzymes, namely, ADAR (also known as ADAR1, human Gene ID 103) and ADARB1 (also known as ADAR2, human Gene ID 104) that are responsible for editing activity. The product from the third locus, ADARB2 (ADAR3, human Gene ID 105) does not have known catalytic activity, despite encoding an intact open reading frame (Melcher et al. 1996; Wang et al. 2019), and is thought to regulate the activity of the other two genes (Oakes et al. 2017) e.g., by competing with other ADARs for substrate (Savva et al. 2012), particularly in the brain (Mladenova et al. 2018). There are two ADAR1 isoforms expressed from the ADAR locus - a larger ADAR p150 isoform expressed from an interferon-inducible promoter due to its consensus ISRE element, and a smaller ADAR p110 isoform (George and Samuel 1999; George et al. 2005). The latter isoform is an N-terminally truncated version of p150 that is constitutively expressed and is predominantly localized to the nucleus (Patterson and Samuel 1995; Pfaller et al. 2011; Samuel 2019). Notably, expression and editing activities of one ADAR family member can be influenced by expression and editing activities of the other ADARs (Cenci et al. 2008; Gallo et al. 2017), including interactions with other components of the innate immunity in viral infections (Athanasiadis 2012). It is the dual role of ADARs as (i) prominent transcriptome regulators in the central nervous system on the one hand (Li and Church 2013), and (ii) as important components of the innate immune response on the other that mechanistically link viral infections with neurologic consequences (Piontkivska et al. 2019).

To complicate things further, there are two types of ADAR editing: (i) a highly selective one, where deamination occurs at a specific site within a transcript, such as the Q/R site of the glutamate receptor subunit GRIA2 (Kawahara et al. 2004), and (ii) a nonselective hyperediting, where multiple A's are edited at once (Bass 2002). Both isoforms of ADAR1 and ADAR2 have been associated with these two types of editing (Lehmann and Bass 2000; Nishikura 2016), although the specific details of editing events – while clearly highly nuanced and spatio-temporaly regulated (e.g., Cruz et al. 2020) – remian to be elucidated. Both types of editing are important in post-transcriptional regulation and transcriptome diversification of the host cells, albeit in mammalian cells the majority of editing events is nonselective hyperediting that occurs within non-coding repetitive regions of mobile elements (Eisenberg and Levanon 2018; Porath et al. 2014; Ramaswami et al. 2012). Yet, in the case of selective editing, seemingly minor changes in a handful of individual codons can result in profound changes in target proteins - such as neural receptors and transporters (Hood and Emeson 2012; Rosenthal and Seeburg 2012) - including their electrophysiological properties, stability, specific recognition sites, and other important features (Nakano and Nakajima 2018). Such editing (or in some cases, lack thereof) leads to a broad array of downstream neurophysiological changes, including neurodevelopmental defects, decreased proliferation, and neuronal death (Gaisler-Salomon et al. 2014; Nishikura 2016). Nonetheless, nonselective hyperediting that often occurs in edited viral genomes can also have major functional and fitness consequences, where indiscriminately introduced nucleotide changes result in missense and nonsense mutations and changes in dsRNA stability that ultimately disrupt the function of viral proteins and genomes. Interestingly, both selective and non-selective ADAR editing has been described in viral genomes; moreover, such editing was shown to have both proviral as well as antiviral consequences (Figure 1) as part of the innate immune response (Athanasiadis 2012; Jayan and Casey 2002; Samuel 2011; Samuel 2019), e.g., through indiscriminate hypermutations of viral sequences (Carpenter et al. 2009; Piontkivska et al. 2016) or highly selective editing of viral RNAs resulting in beneficial mutations or increased translation of viral genes (Casey 2012; Casey and Gerin 1995; Lamers et al. 2019).

As described in the measles (MeV) and dengue (DENV) virus examples below, the pro-/antiviral action is likely not a dichotomy, where editing has either only pro- or antiviral consequences; but rather appears to be a situation-specific outcome that depends on the viral strain and specifics of the host immune response. For example, (presumably deleterious) sequence changes introduced early in the infection by ADAR editing as part of the antiviral defense may be proviral given the selective context imposed by a different arm of the immune response, e.g., in immunocompromised individuals. In the latter category, the most prominent example is ADAR1's ability to block activation of interferon-stimulated protein kinase regulated by RNA (PKR, also known as eukaryotic translation initiation factor 2-alpha kinase 2 [EIF2AK2]), whether through direct binding to PKR or by editing viral dsRNA to prevent activation of dsRNA sensors. This is because activated PKR acts in an antiviral manner by triggering apoptosis, shutting down translation, and enhancing IFN-beta production (McAllister and Samuel 2009; Pfaller et al. 2011; Samuel 2019).

While for many viruses, the molecular details of their interactions with ADARs are only understood at the level of gene expression/viral replication consequences, for many others evidence of A-to-G hyperediting at the sequence level has also been documented (Figure 1). Moreover, the nucleotide neighbor preference of ADARs opens doors to elucidating editing patterns across multiple viruses. Specifically, for editing target A's in the positive strand, those that have A, C, and U as 5' nucleotide neighbor are targeted more frequently than those with 5' G neighbor (Lehmann and Bass 2000; Polson and Bass 1994). Thus, the former category of A's (such as those A's in the dinucleotides AA, UA and CA) can be classified as *Strong* (or *Susceptible* to ADAR editing), and the latter (A's in the GA dinucleotides) as *Weak* (or *Resistant*) ADAR sites, respectively (Bass 2002; Eggington et al. 2011; Samuel 2019). Because ADAR can act on either strand of the dsRNA target, U-harboring residues on the coding strand can also be viewed as potential ADAR sites, i.e., they are A nucleotides on the complementary strand. Thus, coding strand U's can be considered as *Strong* and *Weak* ADAR sites if they have a coding strand 3' A, U, or G, or a 3' C nucleotide neighbor, respectively (Piontkivska et al. 2016).

It is in the context of the prominent role of ADAR editing in the spatio-temporal dynamic regulation of the central nervous system transcriptome (Gallo et al. 2017; Maas et al. 2006; Wahlstedt et al. 2009) that we need to further expand our understanding of interactions between RNA viruses and host ADARs. Beyond the immediate implications for viral fitness or long-term evolutionary consequences in viral genomes, we also need to consider the role of infection-triggered dysregulation of ADAR editing as a factor in neurodevelopmental and neurodegenerative sequelae of viral infections, such as those observed in ZIKV infections (Piontkivska et al. 2017; Piontkivska et al. 2019), or in mouse models of maternal immune activation (Tsivion-Visbord et al. 2020). Sporadic reports indicate that other RNA viruses - including flaviviruses West Nile (WNV) and Japanese encephalitis (JEV), and alphavirus chikungunya (CHIKV) - are also capable of inducing congenital defects and neurological symptoms, or both, in humans and livestock (Al-Fifi et al. 2018; Asnis et al. 2001; Jeha et al. 2003; Oehler et al. 2015; O'Leary et al. 2006; Tsai 2006), although it remains unclear what the underlying mechanisms are or whether such consequences are limited to specific viruses and/or viral clades.

Because of the fundamental role of ADAR editing in animal neural transcriptome diversity and regulation, it is plausible that many more viruses can elicit such neurodevelopmental and/or neurodegenerative consequences, and the lack of documented examples likely reflects lack of attention rather than absence of such instances. The latter phenomenon can be illustrated by Zika virus (ZIKV), which for the first few decades after its discovery was thought to cause benign-to-mild infections (Duffy et al. 2009; Fagbami 1979; Simpson 1964) until it was linked to adverse pregnancy outcomes and birth defects such as microcephaly (Rasmussen et al. 2016), as well as Guillain-Barre syndrome (GBS) (Cao-Lormeau et al. 2016). Subsequently, it was shown that the African strain of ZIKV is as capable of eliciting just as severe fetal pathology as the Asian strains (Udenze et al. 2019), which points to gaps in robust medical surveillance (Majumder et al. 2018). Likewise, long-term follow-up of children born from ZIKV-positive pregnancies shows neurodevelopmental and neurocognitive delays even for children who were asymptomatic and normocephalic at birth (e.g., Bertolli et al. 2020; Pecanha et al. 2020; Stringer et al. 2021), indicating that neurological insult occurred even in the absense of detecable clinical symptoms. Yet, gaps in surveillance include lack of monitoring in the majority of subclinical or completely asymptomatic infections (Franca et al. 2016) that nonetheless are capable of inducing adverse neurodevelopmental outcomes which manifest postnatally (Aragao et al. 2017; Lopes Moreira et al. 2018; Walker et al. 2018; Bertolli et al. 2020), with potentially broad reverberating effects across society (Duttine et al. 2020). Thus, in addition to trying to understand major differences among viral strains (e.g., (Simonin et al. 2017)), we also need to expand our understanding of a breadth of a pathological spectrum that even asymptomatic/subclinical viral infections can cause. Such studies need to focus on the role that infection-mediated changes in ADAR editing – whether dysregulated, or not – play in disease etiology, and whether these changes can act as an environmental factor (i.e., part of the exposome) of neurodegenerative and neurodevelopmental disorders (Finch and Kulminski 2019; Wild 2012).

Regrettably, similar worries exist for the ongoing SARS-CoV-2 pandemic, where the overwhelming of medical systems worldwide diminished the available bandwidth for follow-up of seemingly minor symptoms such as anosmia (Giacomelli et al. 2020) that may in turn signal the presence of subsequent (and more clinically serious) neurological sequelae (Aragao et al. 2020; Pezzini and Padovani 2020). Although the details of links between changes in ADAR editing patterns due to viral infections and subsequent short- or long-term neurological consequences remain to be elucidated, available evidence points to their existence across multiple studies (e.g., Mahic et al. 2017; Tsivion-Visbord et al. 2020). Thus, in this review we explore what is known about ADAR editing across a broad range of viral groups to highlight the breadth of different viral groups that have been shown to experience ADAR editing documented at the level of molecular sequence changes of viral genomes and/or at the level of gene expression or viral replication. We also point out other viral groups that by virtue of their interactions with ADAR-carrying hosts are expected to be edited and thus have the potential to be pathogenic, even if the directly elicited symptoms are subclinical or unknown at this time. We do this in the hope of attracting attention to those pathogens currently considered to be mild(er) and to their interactions with the host immunity, including the role of ADAR editing interactions as a driver of immune escape and/or molecular evolution of these viruses. We would also like to underscore the need to better understand fundamentals of molecular evolution across a broad range of RNA viruses, even those that are thought to be clinically insignificant at the moment. Our work builds upon comprehensive reviews of viral ADAR editing by Tomaselli et al. (2015) and by Pfaller et al. (2021), by expanding a number of discussed RNA viruses with reported ADAR editing and by focusing on the sequence-based evidence where available.

**ADAR editing across RNA virus families**

Figure 1 shows the distribution of documented examples of ADAR editing across multiple families of RNA viruses (excluding retroviruses), arranged in a phylogenetic context, together with their known range of hosts, whether host genomes contain ADAR genes, and indicators of whether ADAR action is pro- or antiviral. Here we use major branches of recently proposed megataxonomy of RNA viruses (Koonin et al. 2020) to primarily help highlight viral groups that have so far received less attention than some other groups with regards to ADAR editing, despite their importance as human or livestock pathogens, such as alphaviruses. Further, we also wanted to point out other viral groups in the global virosphere that do not have editing documented so far, though they likely experience it. Figure 1 shows schematic relationships among groups of RNA viruses, taken from the recent analyses of five major branches based on RdRp shared across these groups and assumed to be monophyletic, despite the high degree of divergence across groups (Wolf et al. 2018) (see also Koonin et al. (2020) and Wolf et al. (2020)). We refer to major groups as per conventions used in Wolf et al. (2018), placing documented examples of ADAR editing at the respective branches/clades. Additional details of what is known about specific ADAR enzymes involved in viral editing, and whether editing leads to pro- or antiviral outcomes are listed in Supplementary Table 1 for each virus, although in many cases these details currently remain unknown.

There are several RNA viruses that have documented instances of ADAR editing, yet are omitted from Figure 1 because they do not share RdRp with other groups and, thus, were not included in the original phylogenetic analyses. These include retroviruses (e.g., HIV-1), replicated via reverse transcriptases, and satellite hepatitis D virus (HDV), which lacks its own RdRp and relies on an obligatory helper such as hepatitis B virus (HBV) envelope protein (Hughes et al. 2011; Lai 2005). Interestingly, the recent discovery of multiple HDV-like viruses in multiple invertebrate and vertebrate species that were not associated with HBV (Chang et al. 2019) and that lack editing sites (Wille et al. 2018) hints at ADAR editing potentially playing a role in driving virus-specific adaptations to particular hosts.

Moreover, it should be noted that A-to-G editing via ADAR enzymes is a phenomenon limited to metazoans, ranging from various invertebrates (from coral and sponges), to *Caenorhabditis elegans*, to insects, to vertebrates, where ADARs contribute to transcriptome diversity and editing of non-coding dsRNAs (Grice and Degnan 2015; Jin et al. 2009; Porath, Knisbacher et al. 2017; Porath et al. 2017). Thus, we expect viruses that infect these groups to experience some form of ADAR editing, if simply due to the presence of viral dsRNA structures. Interestingly, while fungi do not have orthologs of ADAR genes, some fungi exhibit stage-specific A-to-G editing that occurs during sexual reproduction and may be linked to pathogenesis (Liu et al. 2016; Teichert 2018; Teichert et al. 2017; Wang et al. 2016). Similar to plants, fungal genomes lack ADAR orthologs, i.e., no proteins that harbor adenosine deaminase and a dsRNA-binding domain have been identified so far (Teichert 2018; Teichert et al. 2017). It has been proposed that ADAT-like (adenosine deaminase acting on tRNA) enzyme may be responsible (Liu et al. 2016) or that fungi evolved their own class of editing enzymes (Teichert 2018; Teichert et al. 2017), e.g., ones where editing specificity is mediated by small RNAs (Knoop 2011) rather than dsRNA binding domains (Bass 2002). Nonetheless, the mechanism of A-to-G editing in fungi is thought to be distinct from the ADAR-mediated one that we focus on here, and thus, we do not expect fungal viruses to harbor footprints of ADAR editing, or at least not in the form observed in metazoan viruses. Likewise, unless it is discovered that (yet unknown) fungal editors are shared with metazoans, we do not expect animal viruses to experience fungal-mediated A-to-G editing.

Below we summarize what is known about evolutionary consequences of ADAR editing across major RNA viral groups (taxonomic names follow conventions used by Wolf et al. (2018), shown in Figure 1).

## Editing in Bunyavirales

Order Bunyavirales encompasses a broad group of linear negative- or ambi-sense RNA viruses with segmented genomes (Abudurexiti et al. 2019) from a broad range of hosts, from plants to invertebrates to vertebrates (Barr et al. 2021). While a handful of bunyaviruses are already known human pathogens, others share characteristics typical of emergent pathogens such as segmented genomes, persistent infection cycles in vector organisms and broad distribution of hosts (Barr et al. 2021). Evidence of viral ADAR hyperediting has been reported in an attenuated, yet IFN-inducing, clone of Rift valley fever virus (RVFV), where a cluster of A-to-G mutations was identified across multiple sequence variants of its L gene, likely producing an antiviral effect because of diminished viral growth (Suspene et al. 2008). While the study did not measure the activity of ADAR p150 directly, its expression can be expected in the same cell culture that similarly produced hyperediting of measles virus (Suspene et al. 2008).

In lymphocytic choriomeningitis virus (LCMV), a member of the Arenaviridae family, evidence of ADAR hyperediting has been reported within the context of the activated IFN pathway and resultant elevated levels of ADAR p150 isoform (Zahn et al. 2007). Because the majority of reported A-to-G mutations mostly resulted in nonfunctional viral proteins, this study linked antiviral ADAR editing activity with both diminished viral fitness and elevated expression of ADAR p150 in host cells due to IFN activation (Zahn et al. 2007), though some of these changes may contribute to subsequent viral escape in persistent infection (Lewicki et al. 1995). Notably, congenital infections with LCMV, a rodent-borne virus, are associated with both birth defects and pregnancy complications, including congenital hydrocephalus, microcephaly or macrocephaly, and intellectual disabilities (Barton and Hyndman 2000; Barton and Mets 2001). Further, despite frequently being asymptomatic in adult infections, LCMV had been associated with cases of meningitis and GBS, although the mechanisms remain unclear (Barton and Mets 2001; Bonthius 2012).

## Editing in Orthomyxoviridae

The influenza A virus (IAV) is known to generally induce strong immune response, including activation of type I IFNs (Neumann et al. 2021). Interestingly, both the intense cytokine response resulting from a high viral load (referred to as cytokine storm) (de Jong et al. 2006) and cytokine dysfunction, including IFN deficiency (Ciancanelli et al. 2015; Lim et al. 2019), have been associated with the risk of severe influenza (Neumann et al. 2021; Peiris et al. 2004). In other words, disease state can be caused by IFN dysregulation in either direction, whether exaggerated production or the in-born errors leading to IFN deficit (Hernandez et al. 2018; Ryabkova et al. 2020). Of note, impaired IFN responses have been also reported in severe SARS-CoV-2 infections (Bastard et al. 2020; Zhang et al. 2020).

The antiviral role of ADARs had been documented as both a decrease in viral replication (Ward et al. 2011) and observed changes at the nucleotide level, where poor induction of ADAR1 was associated with a decreased number of A-to-G substitutions in the M (matrix) gene of IAV, accompanied by a higher viral load and stronger inflammation (Tenoever et al. 2007). On the other hand, ADAR1 can also act in a proviral manner, where viral growth and neuraminidase activity were reduced in the presence of catalytically inactive ADAR1 construct (de Chassey et al. 2013). Subsequent studies showed that the two ADAR1 isoforms act in an opposite manner, with p110 acting as an antiviral factor, restricting IAV replication, while p150 instead enhances replication because of its ability to suppress IFN-beta and RIG-I-like receptor signaling, the latter of which is thought to be a part of the broader role of p150 in preventing hyperactivation of the innate immune response during viral infections (Heraud-Farlow and Walkley 2020; Vogel et al. 2020).

The dual role of ADARs in modulating IAV replication, whether dependent or independent of editing activity (Vogel et al. 2020), may further vary due to interactions between viral nonstructural NS1 and NS2 proteins and ADARs (de Chassey et al. 2013; Ngamurulert et al. 2009), and between IAV subtypes and/or cell types. For example, ADAR1 was found to be upregulated in H1N1 and H3N2 infections, but downregulated in H7N9, resulting in increased or decreased A-to-G editing in human epithelial cells, respectively, while H5N1 had no effect on the editing (Cao et al. 2018). Interestingly, similarly to human cells, cells of chicken and quail, natural hosts of IAV, also showed no changes in editing for H5N1 infections, which can be attributed to H5N1 suppressing innate immune response, including ADARs (Samy et al. 2016; Suzuki et al. 2009). However, further studies are needed to elucidate differences in editing patterns across different IAV strains and their links to pathogenicity, including surveying animal hosts and even cell cultures used in vaccine production (Suspene et al. 2008).

## Editing in Mononegavirales

Order Mononegavirales encompasses a large and diverse group of nonsegmented linear (-)ssRNA viruses that have a broad range of hosts and includes numerous pathogens of humans and other animals, including measles virus (MeV), Ebola virus (EBOV), human parainfluenza viruses (HPIV), mumps virus (MuV), vesicular stomatitis virus (VSV), as well as plant viruses (Amarasinghe et al. 2019). It also includes Sendai virus (SeV), which is frequently used in mouse disease models (Faisca and Desmecht 2007).

Hyperediting in the measles virus genome was one of the first studied examples of ADAR editing, where clustered A-to-G mutations were identified from persistent MeV infection samples isolated (Cattaneo et al. 1988) even before ADAR enzymes were described (Pfaller et al. 2021). Subsequent studies showed that such hyperediting is due to the action of the IFN-induced ADAR p150 isoform (Suspene et al. 2011). Moreover, ADAR has been shown to play a proviral (and anti-apoptotic) role in the context of measles infection, by inhibiting antiviral pathways such as PKR and interferon regulatory factor IRF-3 (Toth et al. 2009). Editing in MeV is also thought to play a (proviral) role in enabling persistent infections by facilitating viral escape through generation of novel sequence variants (Pfaller et al., 2021; Tomaselli et al., 2015).

While ADAR activation has been shown to suppress apoptosis in acute MeV infection in cell culture, thus acting in a proviral manner (Toth et al. 2009), the impact of editing on the viral sequences per se was not studied, and thus, it remains unclear whether seemingly proviral consequences are due to viral hyperediting and novel mutations or result from editing-independent complex interactions between ADARs and other members of the innate response pathways, including regulators of IFN production (Yang et al. 2014), PKR and IRF-3 (Okonski and Samuel 2013; Toth et al. 2009). The pro- versus antiviral effects of ADAR may likewise be cell-type dependent, when p150 may also contribute to inhibition of viral replication in MeV (Ward et al. 2011), as well as dependent on the viral strains and specifics of experimental ADAR depletion (Tomaselli et al. 2015). However, the antiviral effect of ADAR p150 was shown in other paramyxoviruses, such as Newcastle disease virus (NDV), SeV, and canine distemper virus (CDV) (Ward et al. 2011).

Other known examples of ADAR editing at the sequence level in paramyxoviruses were described in human respiratory syncytial virus (HRSV), where ADAR-generated A-to-G and U-to-C hypermutations were associated with immune escape (Martinez and Melero 2002). Other paramyxoviruses studied include human metapneumovirus (HMPV) (van den Hoogen et al. 2014) and human parainfluenza virus 3 (HPIV3), where only ADAR-like biased transitions were observed in persistent infection (Murphy et al. 1991). Further, localized sequence variation (such as biased hypermutations) in the attenuated mumps virus ($MuV^{JL}$)

vaccine has been attributed to ADAR (and/or APOBEC) action (Chambers et al. 2009). One possibility is that these changes were introduced during vaccine development when a pathogenic strain of MuV was serially passaged in chicken embryo cells (Buynak and Hilleman 1966), where ADAR-driven hypermutation may have generated two distinct mumps isolates that now comprise the $MuV^{JL}$ vaccine (Afzal et al. 1993). Similar hyperediting was observed in some inactivated influenza A vaccines that were grown on chicken embryo fibroblasts (Suspene et al. 2011), attributed to actions of chicken ADAR1 homolog (Herbert et al. 1995). Recent molecular surveillance of MuV in the United States identified a series of U-to-C hypermutations in the small hydrophobic (SH) gene that result in premature stop codons and/or disrupt the canonical stop codon (Stinnett et al. 2020). Because SH acts as a virulence factor, inhibiting apoptosis and innate immune signaling (Stinnett et al. 2020; Xu et al. 2011), these sequence changes illustrate how initially antiviral ADAR action may result in a subsequent proviral consequence of immune escape.

Likewise, evidence of ADAR hyperediting, detected as a cluster of A-to-G (but not U-to-C) mutations within a GP (glycoprotein) gene, was observed when Ebola virus, a filovirus (EBOV, Filoviridae) was passaged in bat cells. Further examination revealed that these mutations accumulated within the same genome strands and were consistent with the ADAR 5' targeting specificity, with an excess of 5' A/U and depletion of 5' G (Whitfield et al. 2020). Notably, these bat cells have higher expression of ADARs than human cells, which may be an endogenous feature of these bats (e.g., Sarkis et al. 2018), because EBOV infection itself did not result in a significant increase of ADAR expression in these cells (Whitfield et al. 2020). No such hyperediting was observed in human cells that had lower ADAR1 levels than bat cells (Whitfield et al. 2020), although clusters of ADAR-mediated U-to-C mutations were observed in some EBOV sequences collected from humans during the 2013–2016 West African epidemic (Dudas et al. 2017; Park et al. 2015; Tong et al. 2015). It should be noted that the region of accumulated hypermutations is a known target of the humoral immune response during infection (Flyak et al. 2016; Ni et al. 2016; Park et al. 2015), but no such antibodies were present during passaging. Thus, these sequence changes - if they were transmitted from a bat to a human - may offer a source of potential pre-existing immune escape mutations, underscoring the need to better understand and to surveil such genomic sequence changes across a broad range of viral reservoir hosts. Indeed, some such clustered changes, localized within the B-cell epitope, were observed in a handful of patient sequences (Dudas et al. 2017; Park et al. 2015), though such changes can also be introduced by a biased RNA polymerase (Park et al. 2015) or be selected through strong immune pressure. Of the currently identified putatively ADAR-hypermutated sequences, they appear to be capable of human-to-human transmission (Dudas et al. 2017; Smits et al. 2015), although they seem not to possess any fitness advantages over other lineages (Smits et al. 2015). Nonetheless, the surveillance of such variants is important, particularly for large-scale outbreaks or epidemics, as some such variants may re-emerge from Ebola virus disease survivors (Whitmer et al. 2018).

ADAR editing was also documented in another filovirus, Marburg virus (MARV), where clusters of U-to-C and A-to-G changes accumulated in a 3' untranslated region of the NP (nucleocapsid) mRNA and targeted sites had 5' A or U nucleotide neighbors, attributed to p150 activity (Shabman et al. 2014). These editing changes appear to regulate translation while also reducing IFN response to the infection (Khadka et al. 2021). While highly pathogenic EBOV and MARV block IFN response and, hence, suppress expression of many interferon-stimulated genes (Basler and Amarasinghe 2009; Katze et al. 2008), it is interesting to note that the less pathogenic (in humans, but not in nonhuman primates) Reston Ebola virus (RESTV) elicits stronger expression of antiviral and immune genes, since it does not inhibit IFN as efficiently (Kash et al. 2006; Ramanan et al. 2011). It is not yet clear what the implications of these differences are to the viral ADAR editing, as ADAR-editing-driven transcript polymorphisms of GP gene have been observed across all ebolaviruses, including RESTV, as well as Cote d'Ivoire (CIEBOV, strain Boniface), Sudan (SEBOV), and Bundibugyo (BEBOV) ebolaviruses in both Vero cells and primary human macrophages (Mehedi 2012). The latter study did not examine in detail the patterns of individual polymorphisms, only the percentage of polymorphic transcripts using the rapid transcript quantification assay. Based on this measure, the extent of editing appeared to vary across cells and different ebolaviruses (Fig. 26 in Mehedi (2012)).

Another member of the Mononegavirales, Borna disease virus (BoDV), appears to rely on host ADARs activity, specifically, editing by ADAR2 (but not the IFN-regulated ADAR p150) to establish persistent infection in the cell nucleus (Yanai et al. 2020). In this study, cells with knockdown ADAR2 had significantly reduced levels of viral RNA, while in the presence of ADAR2 a portion of BoDV sequences showed both synonymous and nonsynonymous A-to-G changes, likely enabling the virus to evade recognition as non-self RNA upon such edits (Yanai et al. 2020).

Interactions with ADARs have also been reported in multiple members of the Rhabdoviridae family. In vesicular stomatitis virus (VSV) infections, reduced levels of ADAR led to reduced viral replication, although ADAR overexpression had no detectable effect on the viral growth (Li et al. 2010). This proviral effect appears to be independent of ADAR editing, driven instead by PKR expression (Gelinas et al. 2011; Nie et al. 2007). It is not known whether rabies virus (RABV), a highly neuroinvasive virus in humans and animals that almost always results in death (Dietzschold et al. 2005; Lyles et al. 2013) experiences ADAR editing. Both RABV and silver haired bat rabies virus (SHBRV), a frequent source of human rabies cases in the US (Faber et al. 2004; Wang et al. 2005), inhibit IFN responses (Brzozka et al. 2005; Faul et al. 2009). Interestingly, attenuated lab-adapted SHBRV strain is capable of inducing a strong IFN response, including expression of ADARs (Niu et al. 2013; Wang et al. 2005), although sequence editing has not been examined. Overall, it appears that because the wild-type SHBRV is sensitive to IFN treatments (Niu et al. 2013), the virus acts to suppress innate immune responses in natural animal infections, and thereby IFN evasion contributes to its pathogenicity.

ADAR editing has been documented in invertebrate viruses as well. For example, clustered A-to-G hypermutations were identified in some sequences of *Drosophila melanogaster* sigma virus (DMelSV), another rhabdovirus that is vertically transmitted (Carpenter et al. 2009). Analysis of ADAR-driven polymorphisms showed that ADAR editing plays an antiviral role in DMelSV infections, with intensity of editing appearing to differ across flies (Piontkivska et al. 2016). Further, ADAR-mediated changes contribute to within- and between-fly DMelSV diversity, acting as one of the driving factors of short- and long-term molecular evolution of the virus, with the virus experiencing selecting pressure to escape. As a result, genomes of DMelSV exhibit under-representation of ADAR-susceptible sites (those with 5' A/U/C neighbor nucleotide) compared to ADAR-resistant sites (those with 5' G) (Piontkivska et al. 2016).

Similar over-representation of A-to-G was found in a related sigmavirus, DImmSV (which infects *Drosophila immigrans*), but not in CCapSV and PAegRV viruses (that infect medflies *Ceratitis capitata*, and speckled wood butterflies *Pararge aegeria*, respectively), indicating that these viruses may have differing dynamic relationships with their insect hosts (Longdon et al. 2017). The likely antiviral action of ADAR homologs in invertebrates has also been documented in mollusks, where various Malacoherpesviridae (dsDNA viruses) infections resulted in upregulation of ADAR genes in bivalves and gastropod taxa, in turn leading to accumulation of A-to-G hypermutations in viral transcripts (Rosani, Bai, et al. 2019; Rosani, Shapiro, et al. 2019). As found in DMelSV, genomes of some malacoherpesviruses also exhibited underrepresentation of ADAR-susceptible sites (Rosani, Bai, et al. 2019), indicating that ADAR editing in invertebrate hosts is also able to play a role in viral molecular evolution.

## Editing in Reoviridae

Reoviruses are known to strongly induce an IFN response (Hood et al. 2014; Pfaller et al. 2021), although studies of ADAR1/2 knockdowns found no impact on ReoV replication (George and Samuel 2011; Ward et al. 2011), potentially because of the protective core-like subvirion particle that shields viral genomes during replication (Pfaller et al. 2021). Interestingly, a study of neonatal mice injected with reovirus T3D into the brains found that despite strong induction of ADAR p150, the majority of surveyed editing substrates exhibited little to no editing changes due to infection, including no detectable changes within 5HT2C (serotonin 2C receptor) transcripts (Hood et al. 2014). Nonetheless, some genes did experience changes in editing, such as cytoplasmic FMR1 interacting protein 2 (Cyfip2), filamin A (Flna), and bladder cancer-associated protein (Blcap) (Hood et al. 2014), indicating that infection-induced changes in editing patterns may be nuanced and possibly influenced by the stage of development. Studies of animal reoviruses, such as that of grass carp reovirus (GCRV) that infects the grass carp (*Ctenopharyngodon idella*), an important freshwater fish, showed that the ADAR1 homolog is activated in response to viral infection, together with other immune response genes (Rao and Su 2015; Yang et al. 2012), implying a role in antiviral response, although its impact on the viral editing remains to be elucidated.

## Editing in Alphavirus Supergroup

The alphaviruses supergroup includes many zoonotic arboviruses that cycle between arthropod insect vectors and a broad range of vertebrate hosts, from fish to mammals, as well as reptiles and birds (Griffin and Weaver 2021; Powers et al. 2012). Human pathogens are divided into a milder group, with viruses causing arthritis and rash, and more serious ones that can cause encephalitis. ADAR editing has been reported in multiple members of this latter group, including rubella virus (RUBV), CHIKV, and VEEV. Genomic sequences of vaccine-derived rubella viruses (iVDRV) isolated from patients with primary immunodeficiency show evidence of ongoing intra-host evolution, where new mutations are continuously introduced in a clock-like manner in these persistent infections (Perelygina et al. 2019). Both APOBEC and ADAR appear to play a role, with ADAR editing serving as a secondary - yet significant - contributor to the mutational spectra relative to APOBEC influence, contributing to hypermutations in both positive and negative strands (Klimczak et al. 2020). Notably, these sequence changes are accompanied by biological changes, with the iVDRV showing distinct cytopathological characteristics from the vaccine virus in cell culture (Perelygina et al. 2019).

Viral replication was enhanced by ADAR overexpression in STAT1$^{-/-}$ fibroblast cells in two alphaviruses, chikungunya virus (CHIKV) and Venezuelan equine encephalitis virus (VEEV) (Schoggins et al. 2011), indicating the proviral impact of ADAR in this context, potentially through modulation of dsRNA-dependent protein kinase (PKR)-mediated stress responses known to inhibit CHIKV replication (Clavarino et al. 2012; Pfaller et al. 2021). However, the effect of ADAR editing on these viral genomes at the nucleotide level has not been studied, including whether ADAR-enhanced replication is associated with changes in viral fitness and pathogenicity. Furthermore, because the extent of IFN production varies among different alphaviruses and host cells, and even among different CHIKV strains (Clavarino et al. 2012; Her et al. 2010), it is plausible that the substitution-inducing (hyperediting) impact of ADARs would vary across strains and/or among hosts.

## Editing in the Flavivirus Supergroup

ADAR editing has been reported in multiple representatives of Flaviviridae, including hepatitis C (HCV) and Zika virus (ZIKV). Antiviral action of A-to-G editing by ADAR1 was first reported in IFN-stimulated Huh7 liver cells, where multiple A-to-G changes, presumed to be deleterious due to lack of replication, were detected. Subsequent siRNA knockdown of ADAR1 (suppressing expression of p150), but not ADAR2, resulted in increased HCV expression (Taylor et al. 2005), indicating the antiviral role of ADAR1 editing in HCV. However, in addition to the direct antiviral impact due to sequence editing, ADAR1 also plays a role in controlling the HCV viral cycle in an editing-independent manner, by suppressing PKR activation (Garaigorta and Chisari 2009; Pfaller et al. 2021). Further, multiple genomic polymorphisms in ADAR1 gene, some nonsynonymous and others with likely transcriptional regulation consequences, have been associated with differences in liver fibrosis severity in patients, including sex differences (Medrano et al. 2017; Pujantell et al. 2018). The molecular mechanisms behind this remain to be elucidated, though, and may be associated with the broader mechanisms of IFN pathways regulation, including a potential role for the type III IFNs (Prokunina-Olsson et al. 2013).

Interactions with ADARs, linking ADAR expression and viral replication, have been reported in multiple other flaviviruses, including bovine viral diarrhea virus (BVDV), dengue virus (DENV), yellow fever virus (YFV), and West Nile virus (WNV). To counteract degradation of hyperedited viral sequences (Scadden and Smith 2001), as an evasion mechanism, the nonstructural NS4A protein of BVDV binds to ADAR1 (Mohamed et al. 2014), likely reducing ADAR's ability to bind and edit dsRNA. However, the specific impact of this binding on sequence changes is not known. On the other hand, binding of the DENV NS3 protein to ADAR appears to promote both ADAR editing activity and viral replication, similar to the interaction between ADAR and NS1 of IAV (de Chassey et al. 2013; Ngamurulert et al. 2009). It should be noted that because the increased editing activity was measured by a reporter construct of hepatitis delta virus (HDV) (de Chassey et al. 2013), the specific editing impacts on the DENV or IAV sequences, and whether these hypermutations are responsible for enhanced replication, remain unclear. In another study DENV replication was found to be influenced by miR-3614-5p that downregulates both ADAR p110 and p150 expression (Diosa-Toro et al. 2017), suggesting that the role of ADAR1 in DENV infectivity may change between early and later stages of infection from proviral to antiviral. This role-switching may also depend on the specific DENV strain, as some of them were shown to differ in their ability to activate (or suppress) the IFN response (Umareddy et al. 2008), including activation of ADAR. Similarly, expression of ADAR appeared to enhance replication of yellow fever virus (YFV) (Schoggins et al. 2011), while that of West Nile virus (WNV) remained unaffected (Schoggins et al. 2015), although, as with DENV, it is not clear whether these effects are mediated by editing of viral sequences.

Another notable example of ADAR's effects on flaviviruses is Zika virus (ZIKV), where several studies independently documented footprints of ADAR editing in ZIKV genomes. Specifically, an analysis of substitution patterns, including frequencies of Weak (i.e., 5' GA) and Strong (i.e., 5' AA, UA, or CA) dinucleotide targets of ADAR editing (Eggington et al. 2011) (which, in turn, can be thought of as Resistant and Susceptible editing targets (Medzhitov et al. 2012)), and spatio-temporal clustering of potential ADAR-driven substitutions showed that ZIKV genomes harbor evidence of long-term evolutionary interactions with the host ADAR editing. These footprints include over-representation of Weak/Resistant ADAR targets at the $2^{nd}$ codon positions, particularly on the positive strand (Piontkivska et al. 2017). Because in these viruses the positive strand acts as both the genome and mRNA, this reflects stronger purifying selection pressure than the one exerted on the negative strand (Piontkivska et al. 2016). We also found underrepresentation of nucleotide polymorphisms at Weak/Resistant ADAR target sites compared to Strong/Susceptible sites, as well as evidence of spatio-temporal clustering of ADAR-editing-driven substitutions (Piontkivska et al. 2017).

Analysis of nucleotide usage biases and nucleotide content in conserved and variable sites showed an association between likely ADAR editing and the presence of secondary RNA structures, including those in minus strand RNA. It had been suggested that the positive RNA strand may get edited by ADARs during pauses in translation due to the presence of rare codons (Khrustalev et al. 2017). While analyses of ZIKV sequences sampled from patients did not allow us to clearly establish whether the identified editing footprints occurred in mammalian hosts, invertebrate hosts, or both (Piontkivska et al. 2017), analysis of ZIKV-infected *Aedes albopictus* mosquitoes showed enrichment of metabolites associated with ADAR editing (Onyango et al. 2020), consistent with observations of the antiviral role of ADAR editing in insect hosts, as we and others showed in DMelSV (Carpenter et al. 2009; Piontkivska et al. 2016). However, cell culture-based experiments showed that - similar to what was observed in DENV and MeV infections - ADAR1 is also able to play a proviral role in early stages of ZIKV infection, likely in an editing-independent manner by suppressing PKR (Zhou et al. 2019).

## Editing in Picornavirales

The number of viruses in the order Picornavirales has been growing rapidly within the past few years, in part due to metagenomics sequencing. This order includes numerous human and animal pathogens, including those from the family Picornaviridae, such as foot-and-mouth disease virus (FMDV), poliovirus and other enteroviruses, hepatitis A virus (HAV), and various rhinoviruses, among others (Rosenfeld and Racaniello 2021; Zell 2018; Zell et al. 2017). Evidence of ADAR editing has been reported in several members of the order, including poliovirus (PV), a human enterovirus that causes poliomyelitis. Specifically, A-to-G and U-to-C hypermutations that led to virulence recovery were observed in vaccine-derived PV sequences (Liu et al. 2015), and exemplified a reverse evolution from a live-attenuated vaccine strain, Sabin 1. The impact of ADAR editing was inferred on the basis of 5'A/U and 3'A di-nucleotide preferences for A or U edits, respectively (Liu et al. 2015). Unlike rubella iVDRV, where editing-mediated mutations appear to result in diminished fitness of resultant viruses that are thus unlikely to infect others (although further studies are needed; (Perelygina et al. 2019)), vaccine-derived polio strains iVDPV revert to neurovirulence in immunodeficient patients and may be transmitted to others (Aghamohammadi et al. 2017; CDC 2012).

Although such events are quite rare (Kew et al. 2002), the fact that neurovirulent reversal occurs within the first 48 hours post-vaccination (Dunn et al. 1990; Minor et al. 1990) potentially supports the role of IFN-activated ADAR editing acting as an antiviral agent that instead results in a proviral outcome. In other words, what appears to be a proviral role of ADAR may be a consequence of a virus benefiting from “blind” cellular machinery that introduces extra mutations in addition to those generated by the virus’s own low-fidelity RdRp that by chance result in increased viral fitness, as opposed to the viral adaptation in response to ADAR. In encephalomyocarditis virus (EMCV), another picornavirus, indirect evidence of ADAR action was observed when endogenous circular RNAs (circRNA) were degraded upon EMCV infection of HeLa cells, similarly to when these cells were treated with poly(I:C), which activates innate immune responses including ADAR (Liu et al. 2019) that then act as antagonists of circRNA production (Ivanov et al. 2015; Pfaller et al. 2021). It should be noted, however, that while these documented molecular interactions can be expected to result in antiviral ADAR action on EMCV, we are not aware of sequence-based evidence of viral editing. Nonetheless, similar to other families, we expect these viruses to experience ADAR editing, although the long-term role of the introduced nucleotide changes remains to be elucidated.

## Editing in Nidovirales

The order Nidovirales encompasses a group of enveloped (+)ssRNA viruses that cause acute and persistent infections in mammals and birds, of which Coronaviridae represents the largest family. This family consists of coronaviruses (CoVs) that cause respiratory, gastrointestinal, and neurological diseases (Murray et al. 1992; Narayanan and Makino 2008; Weiss and Navas-Martin 2005), including CoVs that cause human colds (Myint 1995; Perlman and Masters 2021). The group also includes highly pathogenic viruses causing severe acute respiratory syndrome, namely, SARS, SARS-CoV-2, and MERS-CoV (Middle East respiratory syndrome-related coronavirus).

Interestingly, unlike other RNA viruses, coronavirus genomes encode a highly conserved nonstructural NS14 protein capable of proofreading (Ma et al. 2015; Robson et al. 2020), resulting in a lower mutation rate and a large genome (Holmes 2009; Jenkins et al. 2002). However, evolutionary rates can also be affected by positive selection, e.g., due to tropism switches from bats to humans in SARS (Chinese SARS Molecular Epidemiology Consortium 2004; Hon et al. 2008). Upon infection, CoVs activate PKR and other genes in the IFN signaling pathways as part of the early antiviral response (Cinatl et al. 2004; Perlman and Masters 2021; Sa Ribero et al. 2020). However, CoVs also employ a broad range of strategies to evade IFN responses. These evasion tools are both active and passive, from replication in double-membraned vesicles to prevent recognition by dsRNA sensors (Wolff et al. 2020), to inhibiting IFN activation by antagonizing IRF-3 (Devaraj et al. 2007), to promoting host mRNA degradation (Kamitani et al. 2006), among others (Sa Ribero et al. 2020). Dysregulation of immune responses, including IFN signaling, has been implicated in SARS (Cinatl et al. 2004), MERS (Fehr et al. 2017), and recently in SARS-CoV-2 (causing COVID-19) (Lucas et al. 2020; Park and Iwasaki 2020; Wilk et al. 2020; Xiong et al. 2020). For example, lower levels of ADAR expression in antigen-presenting cells were reported in those with severe COVID-19 pneumonia compared to that from moderate disease samples (Saichi et al. 2021).

Analyses of thousands of recently generated SARS-CoV-2 genomes, from both intra- and inter-patient comparisons, showed that, overall, the pattern of mutations appears to be consistent with that expected from editing by host APOBECs and ADARs (e.g., Azgari et al. 2021; De Maio et al. 2021; Di Giorgio et al. 2020; Klimczak et al. 2020). The majority of changes are C-to-U and are attributed to APOBEC action, although it remains to be determined whether APOBEC1 and/or APOBEC3A are responsible for these edits (Di Giorgio et al. 2020; Klimczak et al. 2020; Ratcliff and Simmonds 2021). Increased C-to-U editing in SARS-CoV-2 has been associated with an enhanced production of inflammatory cytokines in macrophage cells (Kosuge et al. 2020), thereby potentially contributing to pathogenesis, although the corresponding effects of A-to-G changes, including on IFN signaling in COVID-19 patients, remain to be elucidated. Further, an overall lower fraction of A-to-G polymorphisms in SARS-CoV-2 may reflect an underestimate of total editing activity in these datasets, due to the higher efficiency of ADAR hyperediting compared to that of APOBECs, which in turn results in larger disruption of viral fitness and viral propagation (Di Giorgio et al. 2020). Notably, these edits were not distributed uniformly along the genome (De Maio et al. 2021; Di Giorgio et al. 2020), consistent with the clustering observed with ADAR; though this may also reflect competing evolutionary pressures acting on different genomic regions, including potentially episodic and disparate selection pressures due to numerous overlapping reading frames (Hughes et al. 2001; Hughes et al. 2005; Nelson et al. 2020).

Similar excesses of A-to-G and U-to-C changes were observed in SARS and MERS viruses (Fig. 4 in Di Giorgio et al. (2020)). Additional evidence in support of ADAR involvement in observed A-to-G changes came from analyses of bulk sequencing data of SARS-CoV-2-infected Calu-3 cells (Emanuel et al. 2020; Wyler et al. 2021), where significant enrichment of hyperediting events was found, in association with increased ADAR1 expression and, specifically, with elevated Alu editing index (Picardi et al. 2020), a measure of p150 activity (Roth et al. 2019), supporting the role of ADAR in introducing these sequence changes. On the other hand, nanopore-based direct RNA sequencing failed to detect sequences with A-to-G changes in SARS-CoV-2-infected Vero cells (Kim et al. 2020), which may be attributed to the defective IFN response in these cells that are commonly used to grow IFN-sensitive viruses (Ammerman et al. 2008; Desmyter et al. 1968). It should be noted that different CoVs appear to vary in their sensitivity to IFN ( Felgenhauer et al. 2020; Kindler et al. 2013), and thus, may have different fitness and sequence consequences due to ADAR editing.

ADAR editing was also described in infection by another member of the Nidovirales order, an arterivirus porcine reproductive and respiratory syndrome virus (PRRSV). A-to-G changes were identified in host microRNA sequences, where levels of editing were observed to be higher in infected than in uninfected samples (Li et al. 2015), thus potentially affecting microRNA maturation (Tomaselli et al. 2013) or changing target specificity (Ishiguro et al. 2018). While it is not known whether viral PRRSV sequences were edited along with the host microRNAs or what the effect of that might be, accumulating evidence supports involvement of microRNA editing in immune responses, including in viral infections (Contreras and Rao 2012; Marceca et al. 2021; Nachmani et al. 2014). In fact, some viruses - such as various herpesviruses (DNA viruses) - encode and express viral microRNAs of their own, as part of their immune evasion strategy (Fruci et al. 2017; Skalsky and Cullen 2010). An excess of A-to-G changes was also reported in several attenuated PRRSV strains that underwent successive passaging through monkey kidney cells as well as in strains passaged in pigs *in*

*vivo*. These changes were particularly prominent in early passages, indicating that ADAR editing may play a role in changing the virulence of PRRSV (Dong et al. 2019; Li et al. 2015), although the role of specific residue changes in both short-term pathogenicity changes and long-term evolution remains to be elucidated.

## Editing in Caliciviridae

The Caliciviridae family is a family comprised of positive-strand ssRNA viruses and includes noroviruses, major human pathogens that cause acute gastroenteritis, as well as other animal viruses that cause a broad range of diseases across vertebrates, from fish to birds and mammals (Vinje et al. 2019; Wobus and Green 2021). Evidence of A-to-G and U-to-C hyperediting was reported in human norovirus (HuNoV), which has been shown to significantly increase ADAR expression (Lin et al. 2020), likely as events that occur on both strands during replication (Cuevas et al. 2016). Consistent with ADAR editing preferences, 5' nucleotide neighbors of edited residues had significant under-representation of (ADAR-resistant) G's compared to other nucleotides (Cuevas et al. 2016; Lehmann and Bass 2000), similar to what we observed in ZIKV and DMelSV (Piontkivska et al. 2016; Piontkivska et al. 2017). The role of ADAR editing in the molecular evolution of HuNoV is supported by the differences in frequencies of hyperedited sequences between clinical samples and transfected assays, with the former harboring a smaller number of hyperedited genomes likely due to purifying selection (Cuevas et al. 2016), an interpretation which would support an anti-viral role for ADAR in this virus.

Interestingly, similarly to the pattern we detected in DMelSV (Piontkivska et al. 2016), analysis of the distribution of variable and conserved sites among partial capsid sequences of HuNoV isolated from multiple outbreaks (PopSet 158117225; sequences from Ozawa et al. 2007) showed that among A-harboring sites, variable sites were significantly over-represented among ADAR-susceptible ones (those with 5' A, U, or C) (Fisher's two-tailed test, $P$ = 0.0291). Specifically, using multiple alignment sites shared among at least 178 HuNoV sequences, we identified 9 resistant and 10 susceptible A's among conserved A's, versus 6 resistant and 28 susceptible among variable A's (Piontkivska, unpublished), with ADAR susceptibility status defined per randomly chosen representative, EF630492.1 (Hu/GII/143/JPN) of GII/4 cluster associated with the largest number of outbreaks (Ozawa et al. 2007). This pattern of excess of polymorphic A's that are susceptible to ADAR editing, similar to the previously reported pattern in DMelSV (Piontkivska et al. 2016), supports the evolutionary role that ADAR editing plays in this group of viruses and the action of purifying selection, although the specific functional consequences of introduced mutations remain to be elucidated.

## Editing in Other Viral Groups

The phylogenetic tree presented in Figure 1 encompasses very broad groups of RNA viruses; however, it excludes those that do not share RdRp genes, such as hepatitis delta virus (HDV) and retroviruses, such as human immunodeficiency virus (HIV). Indeed, ADAR editing plays a key role in the life cycles of these viruses too. In HDV, site-specific editing by ADAR1 p110 in the nucleus facilitates virion formation by editing a stop codon into a tryptophan codon (at the so-called amber/W site) (Casey 2012; Casey et al. 1992; Hamilton et al. 2010; Pfaller et al. 2021), although the editing efficiency varies among HDV genotypes (Le Gal et al. 2017). Interestingly, examination of sequence polymorphisms within genomes of recently described deltaviruses isolated from birds and mammals identified potential stop codons that experience ADAR editing as signified by reads harboring a minority G nucleotide (Iwamoto et al. 2021). However, because the resultant product appears dysfunctional, it is likely that even if ADAR editing occurs at those sites, it does not contribute to virion production in the same way as it does in the human HDV (Iwamoto et al. 2021).

We refer the reader to excellent comprehensive reviews by Pfaller et al. (2021) and Tomaselli et al. (2015) for extensive descriptions of various effects that ADARs elicit on various retroviruses, including HIV, where both antiviral (e.g., Biswas et al. 2012) and proviral (e.g., Doria et al. 2009) consequences have been extensively documented. However, to keep in line with our primary focus on molecular sequence changes, we will note that ADAR-mediated hyperediting at the sequence level has been described in retroviruses too. For example, ADAR-linked A-to-G hyperediting was reported in two avian retroviruses, Rous-associated virus type 1 (RAV-1) (Felder et al. 1994) and avian leucosis virus (ALV) (Hajjar and Linial 1995). Likewise, hyperediting was detected in human and simian T-cell leukemia viruses (HTLV-2 and STLV-3) in cell culture but not *in vivo*, indicating that it may be a relatively rare event (Ko et al. 2012). Because detectable levels of ADAR1 expression were detected in these blood samples (Ko et al. 2012), it is also possible that the sequencing of only twenty 3DI-PCR products was not sufficient to identify hyperedits. ADAR editing was also shown to play a role in the molecular evolution of equine infectious anemia virus (EIAV), where ADAR-driven A-to-G substitutions enabled the virus to adapt and to become pathogenic in a different species (Tang et al. 2016).

**Conclusions:**

Here we summarize what is known about the evolutionary consequences of ADAR editing across major groups of RNA viruses, highlighting existing gaps in our understanding of the virus-host interactions. Mounting evidence indicates that RNA editing, and ADAR editing in particular, plays a non-trivial role in the molecular evolution of RNA (and other) viruses as a source of novel substitutions. However, for many viral groups, the impact of editing has not been studied at all, while for others, nuances of viral interactions with the host immune response - which can act in a pro- or antiviral manner - remain to be elucidated. Some of these gaps in knowledge, such as whether the editing has been studied at the sequence level, are summarized in Supplementary Table 1. Likewise, in many cited examples the precise nature of viral editing, whether editing a specific codon, or footprints of nonspecific hyperediting, the ADAR isoform responsible, and editing consequences for viral fitness remain unknown. As described in several examples (and conceptualized in Figure 2), the consequences of virus-ADAR interactions, whether they enhance viral replication or diminish it, may be better thought of as a continuous rather than discrete variable, reflecting the variation in the magnitude of measurable impact of editing on viral replication (or fitness). Moreover, whether editing is pro- or antiviral can change during the course of infection. We suggest that editing impact be considered to be dynamic, rather than a binary (mutually exclusive) designation of either proviral or antiviral. Thus, we believe that more attention needs to be devoted to better understanding the spectrum of these virus-ADAR interactions as drivers of the molecular evolution of viruses, particularly in viral groups that are likely to experience ADAR editing but currently lack documented evidence (marked with grey question marks in Figure 1). Particular consideration must be given to temporal and other factors that may facilitate transition between these viral consequences, including the selective or non-selective nature of editing events, possibly at a level of individual transcripts (Prazsák et al. 2018) or across different viral strains, and their impact on pathogenesis. As highlighted in the Supplemental Table 1, such details are often not available even for well-studied viral groups.

While the ongoing pandemic has led to “covidization” of many research areas (Adam 2020), this impact is particularly relevant to infectious diseases (Pai 2020), where interest (and available resources) has been redirected, leaving other pathogens (and underlying pathogenesis mechanisms) under-explored. For example, interest in Ebola virus (as approximated by the number of papers referenced in Pubmed with the terms “Ebola” or “EBOV”) has been waning since its peak in 2015. Similar drop-offs are observed for studies of Zika and hepatitis C viruses, among others. Although the sheer quantity of studies does not necessarily reflect either the quality or breadth of research efforts, the skew in overall numbers may indirectly reflect intensity or types of efforts (such as clinical versus basic mechanisms (Doanvo et al. 2020) and/or focus on (or lack thereof) preparedness and long-term projects (French et al. 2009). Further, emerging viruses other than coronaviruses will certainly continue to appear, such as ongoing animal-to-human infections by various subtypes of avian influenza A virus (WHO 2021).

Thus, understanding the distribution and impact of ADAR editing - as a source of novel mutations, a potential proviral factor, and a cause of changes in host editing resulting in neurological complications - across broad groups of viruses, including in those not currently designated as “pandemic threat,” will serve as an important contribution toward the better handling of future outbreaks and epidemics and will facilitate better diagnostics (and potentially treatment) of novel diseases. Akin to a person looking for keys under the streetlight, we need to broaden our interests to include areas that are not currently illuminated, to extend the analogy. For example, early in the SARS-CoV-2 pandemic, mild neurological symptoms such as “loss of smell” were mostly ignored (e.g., StatNews, March 23, 2020) until it was shown that such seemingly mild symptoms are associated with brain abnormalities, even if transient (Boldrini et al. 2021; Meinhardt et al. 2021; Politi et al. 2020). While anosmia is not particularly surprising for a respiratory virus, if, e.g., it is assumed to result from direct viral damage to the olfactory bulb (Dey et al. 2020), it may be an important clinical symptom to consider in a patient without other upper-respiratory symptoms as a sign of dysregulated inflammatory response (Han et al. 2020), including potential dysregulation of ADAR editing. Indeed, the growing body of evidence points to a wide array of neurological symptoms in COVID-19 patients, even in those with a mild or moderate course of the disease (Beghi et al. 2021; Ellul et al. 2020), including evidence of a long-term involvement of the central and peripheral nervous system, such as GBS (Abu-Rumeileh et al. 2021; Gupta et al. 2020; Palaiodimou et al. 2021). Other post-COVID complications include potentially permanent cognitive impairments that are not linked to severe disease or ICU stay (Damiano et al. 2021; Del Brutto et al. 2021), elevated risk of Parkinson's disease (PD) development (Brundin et al. 2020), or unmasking of preclinical PD (Merello et al. 2021). Dysregulation of neuroinflammatory pathway following viral infections – such as IAV and WNV - have been associated with development of PD, although specific underlying mechanisms remain to be elucidated (Henry et al. 2010; Limphaibool et al. 2019). Changes in ADAR editing has been described in multiple neurodegenerative and neuropsychiatric disorders (e.g., Gaisler-Salomon et al. 2014; Gardner et al. 2019; Khermesh et al. 2016; Plonski et al. 2021). While we are still in the early stages of understanding these nuanced and dynamic changes and their relationships with viral infections, as described in this review, multiple viruses, including SARS-CoV-2, are capable of activating ADARs and potentially dysregulating the normal editing patterns. Better understanding of virus-mediated editing changes in host transcriptomes may offer insights for the diagnostics and treatment of COVID-19 patients, including those with so-called long COVID, as well as those left with unexplained neurological symptoms from prior viral infections. Understandably, many medical systems are overwhelmed with the sheer number of COVID-19 patients at the moment. However, for those who can, collecting - and sharing - transcriptome-wide sequencing data that can be used for inferring editing patterns, jointly with clinically relevant information, will help us better understand the sequelae of COVID-19, as well as other viral diseases.

**Figure legends:**

Figure 1. Documented cases of ADAR editing among major RNA virus groups, indicating whether sequence-based evidence (reflecting A-to-I/A-to-G or U-to-C substitutions; marked with superscript $^{A/G}$) are available for individual viruses. Phylogenetic relationships are given per Wolf et al. (2018), grouping RNA viruses with RNA-dependent RNA polymerases (RdRp) into major 5 clades (designated with vertical bars). Host distribution and whether these hosts harbor ADAR genes (marked with boxed checkmarks) is also shown for each major branch. Host icons represent bacteria and/or protozoa; plants; fungi; invertebrates; vertebrates; human pathogens (full circle marks the relevant host(s)). Pro- or antiviral action of ADARs is indicated with up or down arrows, reflecting the position of relevant studies cited in this review. Circled question marks designate viral groups that infect hosts with functional ADAR genes, and hence, can be expected to experience ADAR editing, although the evidence for or against it is currently lacking.

Abbreviations: ADARs, adenosine deaminases acting on RNA; BEBOV, Bundibugyo ebolavirus; BoDV, Borna disease virus; BVDV, bovine viral diarrhea virus; CDV, canine distemper virus; CHIKV, chikungunya virus; CIEBOV, Cote d'Ivoire ebolavirus; DENV, dengue virus; DImmSV, *Drosophila immigrans* sigma virus; DMelSV, *Drosophila melanogaster* sigma virus; EBOV, Ebola virus; EMCV, encephalomyocarditis virus; GCRV, grass carp reovirus; HCV, hepatitis C virus; HIV, human immunodeficiency virus; HMPV, human metapneumovirus; HPIV3, human parainfluenza virus 3; HRSV, human respiratory syncytial virus; HTLV, human T-cell leukemia virus; HuNoV, human norovirus; IAV, influenza A virus; LCMV, lymphocytic choriomeningitis virus; MARV, Marburg virus; MERS, Middle East respiratory syndrome-related coronavirus; MeV, measles virus; MuV, mumps virus; NDV, Newcastle disease virus; PRRSV, porcine reproductive and respiratory syndrome virus; PV, poliovirus; ReoV, reovirus; RESTV, Reston ebolavirus; RUBV, rubella virus; RVFV, Rift Valley fever virus; SARS, severe acute respiratory syndrome-related coronavirus; SARS-CoV-2, SARS coronavirus 2; SEBOV, Sudan ebolavirus; SeV, Sendai virus; SHBRV, silver haired bat rabies virus; VEEV, Venezuelan equine encephalitis virus; VSV, vesicular stomatitis virus; WNV, West Nile virus; YFV, yellow fever virus; ZIKV, Zika virus.

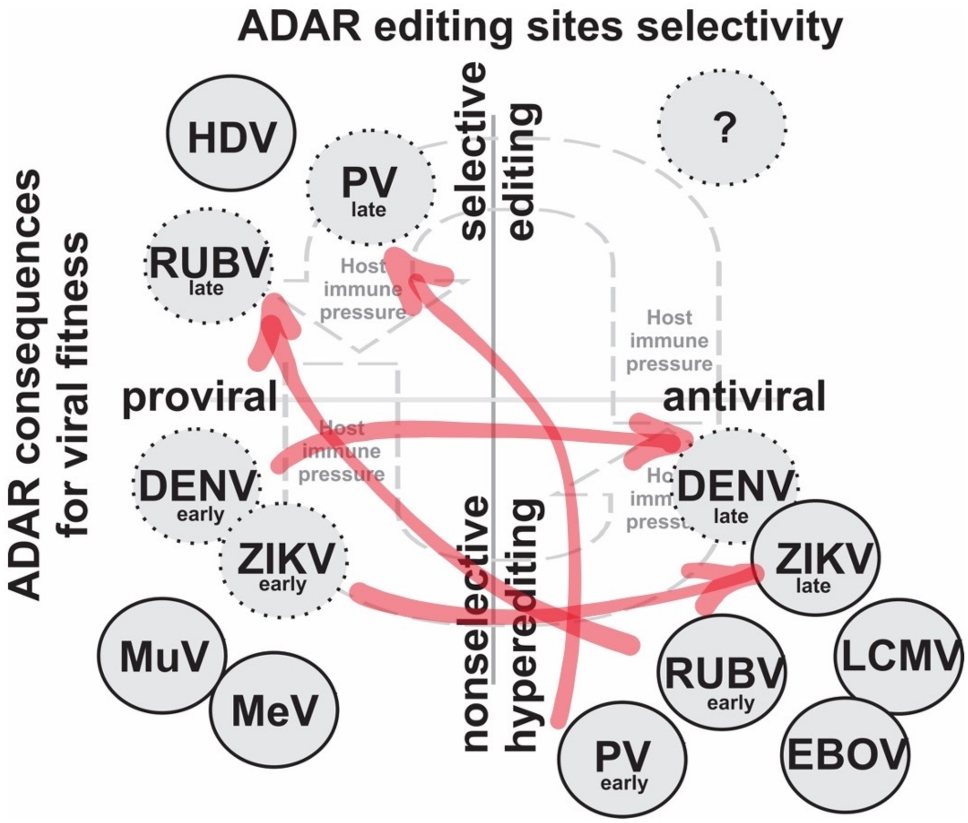

Figure 2. Conceptual framework of intersecting axes of ADAR editing selectivity (ranging from nonselective hyperediting to selective editing) and fitness consequences of virus-ADAR interactions, ranging from proviral to antiviral. We propose that the fitness consequences for specific viruses be considered as continuous rather than discrete (binary) variable due to variation in the magnitude of measurable impact of editing on viral replication (or fitness). Likewise, the direction the impact, whether pro- or antiviral, can dynamically change during the course of infection and/or between short- and long-term evolutionary consequences. Thus, the same virus may be categorized into different quadrants depending on the circumstances, including stage of infection or specifics of host immune response. For example, DENV may experience proviral effects, such as enhanced replication, due to interactions with ADARs early in the infection (de Chassey et al. 2013), followed by subsequent antiviral consequences due to hyperediting; this switch is depicted by the semi-transparent red arrow (Diosa-Toro et al. 2017). Solid circles designate virus-ADAR interactions with documented sequence-based ADAR editing; dotted lines mark non-sequence-based interactions. Viral names are abbreviated as in Figure 1.

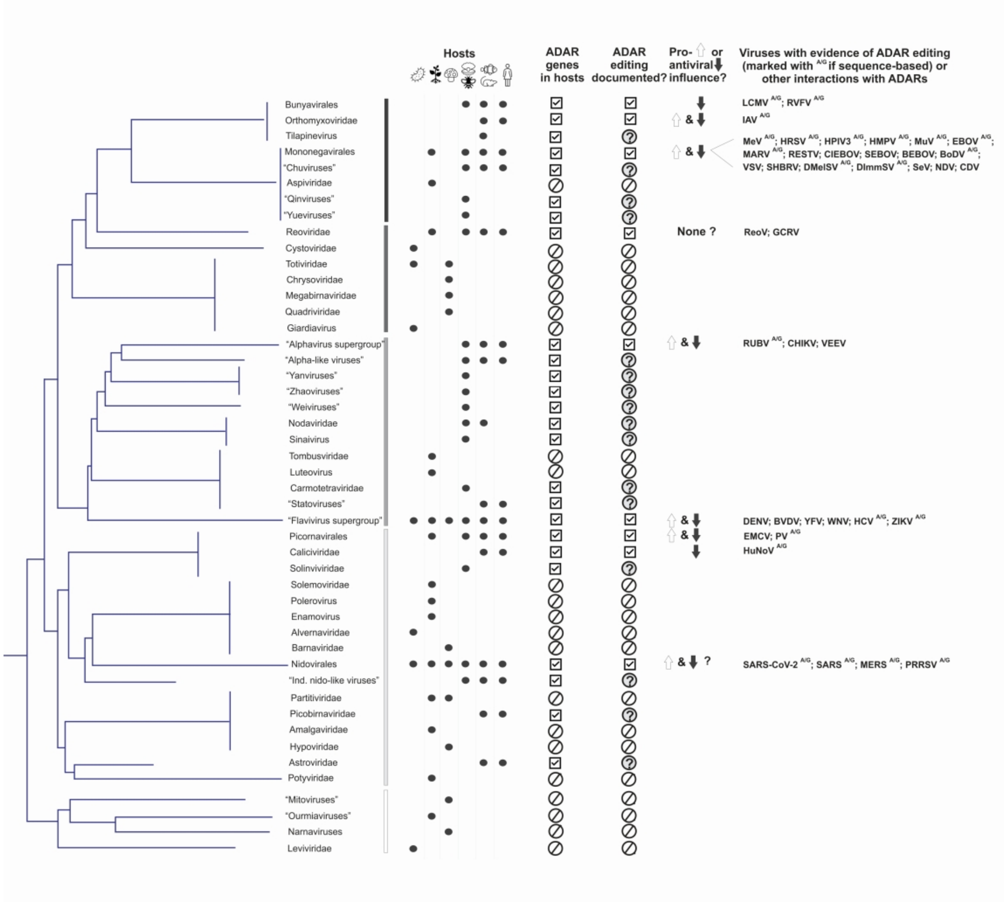

Figure 1. Documented cases of ADAR editing among major RNA virus groups, indicating whether sequence-based evidence (reflecting A-to-I/A-to-G or U-to-C substitutions; marked with superscript A/G) are available for individual viruses. Phylogenetic relationships are given per Wolf et al. (2018), grouping RNA viruses with RNA-dependent RNA polymerases (RdRp) into major 5 clades (designated with vertical bars). Host distribution and whether these hosts harbor ADAR genes (marked with boxed checkmarks) is also shown for each major branch. Host icons represent bacteria and/or protozoa; plants; fungi; invertebrates; vertebrates; human pathogens (full circle marks the relevant host(s)). Pro- or antiviral action of ADARs is indicated with up or down arrows, reflecting the position of relevant studies cited in this review. Circled question marks designate viral groups that infect hosts with functional ADAR genes, and hence, can be expected to experience ADAR editing, although the evidence for or against it is currently lacking. Abbreviations: ADARs, adenosine deaminases acting on RNA; BEBOV, Bundibugyo ebolavirus; BoDV, Borna disease virus; BVDV, bovine viral diarrhea virus; CDV, canine distemper virus; CHIKV, chikungunya virus; CIEBOV, Cote d'Ivoire ebolavirus; DENV, dengue virus; DImmSV, Drosophila immigrans sigma virus; DMelSV, Drosophila melanogaster sigma virus; EBOV, Ebola virus; EMCV, encephalomyocarditis virus; GCRV, grass carp reovirus; HCV, hepatitis C virus; HIV, human immunodeficiency virus; HMPV, human metapneumovirus; HPIV3, human parainfluenza virus 3; HRSV, human respiratory syncytial virus; HTLV, human T-cell leukemia virus; HuNoV, human norovirus; IAV, influenza A virus; LCMV, lymphocytic choriomeningitis virus; MARV, Marburg virus; MERS, Middle East respiratory syndrome-related coronavirus; MeV, measles virus; MuV, mumps virus; NDV, Newcastle disease virus; PRRSV, porcine reproductive and respiratory syndrome virus; PV, poliovirus; ReoV, reovirus; RESTV, Reston ebolavirus; RUBV, rubella virus; RVFV, Rift Valley fever virus; SARS, severe acute respiratory syndrome-related coronavirus; SARS-CoV-2, SARS coronavirus 2; SEBOV, Sudan ebolavirus; SeV, Sendai virus; SHBRV, silver haired bat rabies virus; VEEV, Venezuelan equine encephalitis virus; VSV, vesicular stomatitis virus; WNV, West Nile virus; YFV, yellow fever virus; ZIKV, Zika virus.

325x293mm (300 x 300 DPI)

232x190mm (120 x 120 DPI)

## Supplementary Material

evab240_Supplementary_Data

