# Supplementary material for: ADAR Editing in Viruses: An Evolutionary Force to Reckon with
Source: Genome Biol Evol. 2021 Oct 25;13(11):evab240. doi: 10.1093/gbe/evab240 (PMC8586724; doi:10.1093/gbe/evab240)
Supplement: evab240_Supplementary_Data [file evab240_supplementary_data.pdf]

Supplementary Table 1. Overview of evidence of ADAR editing in viral and/or host sequences, for viruses listed in Figure 1. Multiple gaps in knowledge regarding the mechanism and consequences of ADAR editing are highlighted using the following designations: Grey cells indicate (i) viruses for which sequence-based editing, editing in the host, *etc* was not reported, and (ii) those for which both pro- and antiviral action of ADAR were reported, and thus further studies are needed to elucidate these roles, or (iii) editing impact is unknown. Bolded **ADAR/ADAR2** indicates studies where the exact isoform of ADAR1, p150 or p110, was not reported, or requires further studies of its involvement. "Pro/antiviral ?" designation refers to examples where presumed antiviral ADAR hyperediting was observed to result in immune escape, although further studies are needed to establish whether all cases of hyperediting lead to escape. Moreover, in cases where evidence of ADAR involvement comes from non-sequence-based studies, it is unknown whether such involvement comes from editing or editing-independent actions only, or a combination of both.

Virus	If ADAR editing is inferred from viral sequence-based evidence (marked with AG on Figure 1)	Evidence of hyperediting of viral sequences	Evidence of editing of the host sequences	Reported direction of ADAR action effect (i.e., pro – or antiviral if viral replication is enhanced or suppressed, respectively)	Which ADAR gene is reportedly involved	References
RVFV	Yes	Yes	Not known	Antiviral	ADAR150	(Suspene et al. 2008)
LCMV	Yes	Yes	Not known	Antiviral	ADARp150	(Zahn et al. 2007)
IAV	Yes	Yes	Yes (Cao et al. 2018)	Both antiviral and proviral consequences reported; editing varies among strains (Cao et al. 2018)	ADARp110, ADARp150	(Ward et al. 2011; Tenoever et al. 2007; de Chassey et al. 2013)
MEV	Yes	Yes	Yes (Suspene et al. 2008)	Both antiviral and proviral consequences reported; editing varies among strains (Tomaselli et al. 2015)	ADARp150	(Cattaneo et al. 1988; Suspene et al. 2011; Toth et al. 2009; Ward et al. 2011)
NDV	No	No	Not known	Antiviral	ADARp150	(Ward et al. 2011)
SEV	No	No	Not known	Antiviral	ADARp150	(Ward et al. 2011)
CDV	No	No	Not known	Antiviral	ADARp150	(Ward et al. 2011)
HRSV	Yes	Yes	Not known	Pro/antiviral ?	**ADAR**	(Martinez and Melero 2002)
HMPV	Yes	Yes	Not known	Pro/antiviral ?	**ADAR**	(van den Hoogen et al. 2014)
HPIV3	Yes	Yes	Not known	Pro/antiviral ?	**ADAR**	(Murphy et al. 1991)
MuV	Yes	Yes	Not known	Pro/antiviral ?	**ADAR**	(Chambers et al. 2009; Stinnett et al. 2020)
EBOV	Yes	Yes	Not known	Pro/antiviral ?	**ADAR**	(Whitfield et al. 2020)
MARV	Yes	Yes	Not known	Antiviral	ADARp150	(Shabman et al. 2014)
RESTV	No	Yes	Not known	Unknown	**ADAR**	(Mehedi 2012)
CIEBOV	No	Yes	Not known	Unknown	**ADAR**	(Mehedi 2012)
SEBOV	No	Yes	Not known	Unknown	**ADAR**	(Mehedi 2012)

BEBOV	No	Yes	Not known	Unknown	**ADAR**	(Mehedi 2012)
BoDV	Yes	Yes	Not known	Proviral	ADAR2	(Yanai et al. 2020)
VSV	No	Not reported	Not known	Proviral; editing varies by cell types (Tomaselli et al. 2015)	**ADAR**	(Li et al. 2010; Gelinas et al. 2011; Nie et al. 2007)
SHBRV	No	Not reported	Not known	Antiviral	**ADAR**	(Niu et al. 2013; Wang et al. 2005)
DMelSV	Yes	Yes	Not known	Antiviral	**ADAR**	(Carpenter et al. 2009; Piontkivska et al. 2016)
DImmSV	Yes	Yes	Not known	Unknown	**ADAR**	(Longdon et al. 2017)
CCapSV	Evidence of lack of editing	No evidence of hyperediting	Not known	None	Not applicable	(Longdon et al. 2017)
PAegRV	Evidence of lack of editing	No evidence of hyperediting	Not known	None	Not applicable	(Longdon et al. 2017)
ReoV	Yes, but not detected	No evidence of hyperediting	Yes (Hood et al. 2014)	None	ADARp150, **ADAR2**	(Hood et al. 2014; George and Samuel 2011; Ward et al. 2011)
GCRV	No	No	Not known	Antiviral	**ADAR**	(Rao and Su 2015; Yang et al. 2012)
RUBV	Yes	Yes	Not known	Antiviral	**ADAR**	(Perelygina et al. 2019; Klimczak et al. 2020)
CHIKV	No	No	Not known	Proviral	**ADAR**	(Schoggins et al. 2011)
VEEV	No	No	Not known	Proviral	**ADAR**	(Schoggins et al. 2011)
HCV	Yes	Yes	Not known	Antiviral	ADARp150	(Taylor et al. 2005)
BVDV	No	No	Not known	Antiviral	**ADAR**	(Mohamed et al. 2014)
DENV	No	No	Not known	Both antiviral and proviral consequences reported; editing varies among strains (Diosa-Toro et al. 2017)	**ADAR**	(de Chassey et al. 2013; Ngamurulert et al. 2009)
YFV	No	No	Not known	Proviral	**ADAR**	(Schoggins et al. 2015)
WNV	No	No	Not known	Possibly none	**ADAR**	(Schoggins et al. 2015)
ZIKV	Yes	Yes	Yes, based on metabolites (Onyango et al. 2020)	Both antiviral and proviral consequences reported (Zhou et al. 2019)	**ADAR**	(Piontkivska et al. 2017; Khrustalev et al. 2017; Zhou et al. 2019)
EMCV	No	No	Not known	Antiviral	**ADAR**	(Liu et al. 2019)
PV	Yes	Yes	Not known	Antiviral	**ADAR**	(Liu et al. 2015)

SARS-CoV-2	Yes	Yes	Yes (Emanuel et al. 2020; Wyler et al. 2021)	Antiviral	**ADAR**	(Azgari et al. 2021; De Maio et al. 2021; Di Giorgio et al. 2020; Klimczak et al. 2020)
SARS	Yes	Yes	Not known	Antiviral	**ADAR**	(Di Giorgio et al. 2020)
MERS	Yes	Yes	Not known	Antiviral	**ADAR**	(Di Giorgio et al. 2020)
PRRSV	Yes	Yes	Yes (Li et al. 2015)	Antiviral	**ADAR**	(Dong et al. 2019; Li et al. 2015)
HuNoV	Yes	Yes	Not known	Antiviral	**ADAR**	(Cuevas et al. 2016)
